# Supplementary material for: Quantitative Proteomics of Medium-Sized Extracellular Vesicle-Enriched Plasma of Lacunar Infarction for the Discovery of Prognostic Biomarkers
Source: Int J Mol Sci. 2022 Oct 1;23(19):11670. doi: 10.3390/ijms231911670 (PMC9569577; doi:10.3390/ijms231911670)
Supplement: Supplementary file 1 [file ijms-23-11670-s001.zip › SI_Revised_Final.pdf]

Supplemental Information

Quantitative Proteomics of Medium-Sized Extracellular Vesicle-Enriched Plasma of Lacunar Infarction for the Discovery of Prognostic Biomarkers

Arnab Datta[1,2*], Christopher Chen[3,4], Yong-Gui Gao[2], Siu Kwan Sze[2,5*]

[1] Laboratory of Translational Neuroscience, Division of Neuroscience, Yenepoya Research Center, Yenepoya (Deemed to be University), University Road, Deralakatte, Mangalore 575018, Karnataka, India
[2] School of Biological Sciences, Nanyang Technological University, 60 Nanyang Drive, Singapore 637551
[3] Memory, Aging and Cognition Centre, National University Health System, Singapore
[4] Department of Pharmacology, Yong Loo Lin School of Medicine, National University of Singapore, Singapore
[5] Department of Health Sciences, Faculty of Applied Health Sciences, Brock University, 1812 Sir Isaac Brock Way, St. Catharines, ON L2S 3A1, Canada

*Corresponding author

*Arnab Datta, PhD
Laboratory of Translational Neuroscience,
Division of Neuroscience, Yenepoya Research Center,
Yenepoya (Deemed to be University), University Road, Deralakatte,
Mangalore 575018, Karnataka, India
Tel: (+91) 824-220-4668, Fax: (+91) 824-220-3943
Email: arnabdattaju@gmail.com , arnabdatta@yenepoya.edu.in
ORCID: 0000-0002-9220-7812

*Siu Kwan SZE, PhD
Department of Health Sciences, Faculty of Applied Health Sciences,
Brock University, 1812 Sir Isaac Brock Way,
St. Catharines, ON L2S 3A1, Canada
Tel: (+1) 905 688 5550 X 6098
Email: nsze@brocku.ca
ORCID: 0000-0002-5652-1687

Index

Supplemental Methods

Supplemental Results

Supplemental Table S1. Protein content-based MEV characterization as per MISEV2018 guidelines

Supplemental Table S2. Comparative list of EV markers detected in common between MEV and SEV fractions

Supplemental Table S3. Number of aberrantly altered proteins in various outcome groups based on different filtering criteria

Supplemental Table S4. Proteome-wide correlation analysis in between three different LACI outcome groups

Supplemental Table S5. Summary of Chi-square analysis results based on GO-terms

Supplemental Table S6. Demographic Characteristics of the Patient Population Stratified by the Outcome Measures

Supplemental Figure S1. Frequency histogram showing the relative distribution of log2 ratios in three different LACI groups: (A) NAO, (B) RVE, and (C) CD, when compared to HC.

Supplemental Methods

Sample Collection and Clinical Information

The plasma samples were obtained from patients with a nondisabling ischemic stroke who were recruited at the Singapore General Hospital between 1999 and 2005 for the cognitive sub-study of the ESPRIT (ESPRIT-cog). Detailed methodology of ESPRIT and ESPRIT-cog including the exclusion criteria have been reported previously [1-4]. Briefly, the patients were eligible if they were within 6 months of a nondisabling ischemic stroke (grade ≤ 3 on the modified Rankin scale (mRS)) of presumed arterial origin [5]. All patients were randomized to either aspirin (100 mg/day) or aspirin combined with dipyridamole (75-450 mg/day). The control plasma was collected from non-stroke subjects at the same site during 2004 - 2006. EDTA was used as the anti-coagulant during the processing of blood samples. The exclusion criteria were: a possible cardiac source of embolism, high-grade carotid stenosis for which carotid endarterectomy or endovascular treatment was planned, moderate to severe leukoaraiosis on brain imaging (for randomization into anticoagulation), any blood coagulation disorder, any contraindication for aspirin or dipyridamole, and a limited life expectancy [3].

A neuropsychological test battery was used to determine the cognitive status of the patients during baseline and subsequent follow-up visits. Diagnoses of dementia were made according to the DSM-IV criteria [6]. Any patients who were not demented at the baseline were included in this study. Details of the procedure have been reported earlier [3].

Baseline Risk Factors

Risk factor information was collected at baseline. Stroke subtype was classified according to the Oxfordshire Community Stroke Project as total anterior circulation infarct, partial anterior circulation infarct, posterior circulation infarct, or LACI [7]. Vascular risk factor data, such as age, diabetes mellitus status, hypertension, hyperlipidemia, smoking status, ischemic heart disease, peripheral artery disease, as well as past history of stroke, angina and myocardial infarction were obtained verbally from the patient and confirmed with hospital records.

Experimental Design Guided by Outcome Measures

Of the 458 patients enrolled in the Singapore General Hospital's site of the ESPRIT trial, 26 (6%) refused to provide blood samples and were excluded from the study. Hence, 432 consented to participate in the ESPRIT-Cog sub study. Of these 432 patients, 275 (64%) had LACI, which was the population of interest for the current study. Further, 10 were excluded as they had dementia at baseline. Several patients had insufficient plasma samples or visible hemolysis or dropped out during the follow up period were therefore excluded. Representative 45 (45/265, 17%) LACI patients were selected for this discovery proteomics study. Notably, there were no differences in demographic characteristics between these 45 and remaining 220 LACI patients of this cohort (data not shown).

The experimental design is depicted in **Figure 1 (main text)**. The LACI patients were followed up annually for up to 5 years (median follow up, 3 years; interquartile range, 2 years) to monitor for the occurrence of any vascular event or for change in the baseline cognitive status. Strokes, peripheral artery disease, intracranial bleeds, and any cardiac ischemia (stable and unstable angina, myocardial infarctions) or deaths from any of the above were considered to be a recurrent vascular event. Any LACI patient having a recurrence of vascular event during the follow-up period was included in the group called "recurrent vascular event" (RVE). The patients whose cognitive status declined from the respective baseline status during the course of the prospective study had been assigned to the "cognitive decline" (CD) group. Patients who did not suffer a recurrent vascular event or cognitive decline during this period were grouped as "LACI, no adverse outcome" (NAO). Accordingly, plasma samples of 45 LACI patients were divided into three groups based on the outcome variables (LACI – NAO, n = 19; LACI – RVE, n = 11; LACI – CD but no RVE, n = 15). The control group (healthy control, HC) had 17 subjects who never had a stroke or cancer and were cognitively normal at the baseline. The plasma samples from four groups were pooled group-wise to obtain four sets of pooled plasma samples for proteomics processing.

The demographic characteristics, baseline risk factors and cognitive classifications of the study population stratified by outcome measures and control group are summarized in Supplemental Table 6. The average age of the recruited subjects was 61 ± 10 years; 55% were males and 92% were Chinese. No significant difference was observed between three groups of LACI patients in terms of most of the demographic variables and baseline risk factors except 'gender' ($H(2) = 11.86$, $p = 0.003$) and 'smoking' ($H(2) = 7.276$, $p = 0.026$).

Electrostatic Repulsion and Hydrophilic Interaction Chromatography (ERLIC)

The combined iTRAQ sample was desalted by Sep-Pak C18 SPE cartridges (Waters, Milford, MA, USA). A modified ERLIC with volatile salt-containing buffers was adopted [8]. The dried iTRAQ-labeled peptides were reconstituted in 200 µl of Buffer A (10 mM NH_4HCO_2, 85% ACN, 0.1% formic acid (FA)) and fractionated using a PolyWAX LP column (200 × 4.6 mm; 5 µm; 300 A°) (PolyLC, Columbia, MD, USA) on a Prominence HPLC system (Shimadzu, Kyoto, Japan) in a 65 min gradient with Buffer B (30% ACN, 0.1% FA). The HPLC gradient was composed of 100% buffer A for 10 min; 0–25% buffer B for 35 min; then 25–100% buffer B for 10 min; followed by 100% buffer B for 10 min. The chromatogram was recorded at 280 nm. Eluted fractions were collected in every 1 min, and then pooled into 30 fractions depending on the peak intensities, before drying them in a vacuum centrifuge. They were stored at -20°C till MS analysis.

MS Raw Data Analysis

The spectral data acquisition was performed using the Analyst QS 2.0 software (Applied Biosystems, Foster City, CA). ProteinPilot Software 3.0, Revision Number: 114,732 (Applied Biosystems) was used for peak list generation, protein identification and quantification against the Uniprot human database (191,242 sequences, downloaded on 10 March 2012). A

concatenated target-decoy database search strategy was also employed to estimate the false discovery rate (FDR). FDR was obtained by calculating the percentage of decoy matches among total matches.

FDR = 2.0 × decoy_hits/total_hits

Total hits = Target database (forward) + Decoy database (reverse)

The user defined parameters of the software were configured as described previously with minor modifications (ID Focus: biological modification). The ProteinPilot software employed Paragon (version 3.0.0.0, 113,442) and Pro Group algorithm for the peptide identification and isoform-specific quantification respectively. The iTRAQ reporter ion areas under the curve for individual peaks in a MS spectrum were used for the calculation of peptide-level iTRAQ ratios. Peptides with missing iTRAQ reporter is not used in iTRAQ reporter ratio calculation, and no quantitative information is generated. The protein ratios were calculated from the peptide-level iTRAQ ratios of confidently detected unique peptides after averaging them. Details of the quantification algorithm can be found in the supplier's manual. The resulting data set were auto bias-corrected to get rid of any variations imparted due to the unequal mixing during combining different labeled samples. Subsequently background correction was also performed to eliminate any background ion signal due to nontarget peptides, coeluting with the target peptide.

Supplemental Results

Supplemental Table S6

Table S6. Demographic Characteristics of the Patient Population Stratified by the Outcome Measures#

Characteristic N (%)	No adverse outcome (N = 19)	Recurrent vascular events (stroke + MI) (N = 11)	Cognitive decline (no recurrent vascular events) (N = 15)	Healthy control (N = 17)
Age, Mean (SD) †	61 (9)	65 (10)	66 (9)	56 (9)
**Sex, Male**	17 (90)	8 (73)	5 (33)	4 (26)
Ethnicity, Chinese	17 (90)	8 (73)	15 (100)	17 (100)
Delay to Blood Draw in Days, Median (IQR)*	32.5 (83)	41.5 (80)	59 (68)	NA
Diabetes mellitus	7 (37)	2 (18)	7 (47)	6 (35)
Hypertension	11 (58)	9 (82)	12 (80)	10 (59)
Previous stroke	0 (0)	3 (27)	4 (27)	None
Hyperlipidemia	8 (42)	4 (36)	9 (60)	10 (59)
**Ever smoker**	5 (26)	6 (55)	1 (7)	2 (12)
Previous ischemic heart disease	2 (11)	2 (18)	1 (7)	
Previous myocardial infarction	0 (0)	0 (0)	1 (7)	3 (18)
Previous angina	2 (11)	2 (18)	0 (0)	
Previous peripheral artery disease	0 (0)	0 (0)	0 (0)	None
Baseline cognitive classification				
NCI	13 (68)	2 (18)	8 (53)	
CIND-mild	4 (21)	6 (55)	6 (40)	None
CIND-moderate	3 (16)	3 (27)	1 (7)	

All values are reported as: N (%), where N indicates the number of observations. †Values are expressed as Mean (±standard deviation). *Values are expressed as Median (inter quartile range). NCI, no cognitive impairment; CIND, cognitive impairment no dementia. # Nonparametric Kruskal-Wallis H Test was used for comparing ordinal variables such as demographic characteristics, baseline rick factors and cognitive status between three LACI groups. Except 'gender' ($H(2) = 11.86$, $p = 0.003$) and 'smoking' ($H(2) = 7.276$, $p = 0.026$), other variables are not significantly different between three LACI groups.

Supplemental Figure S1

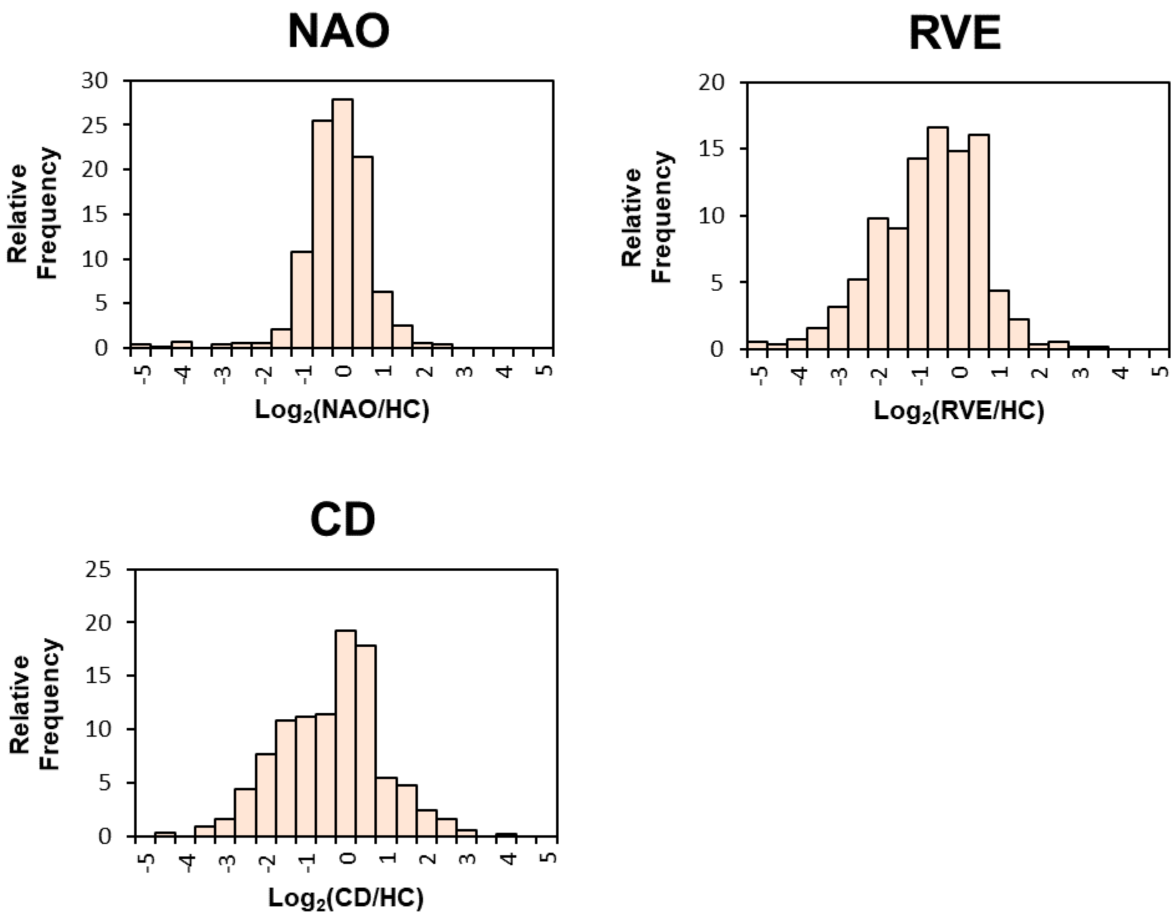

Figure S1. Frequency histogram showing the relative distribution of log2 ratios in three different LACI groups: (A) NAO, (B) RVE, and (C) CD, when compared to HC. The relative frequency refers to the percentage of protein hits with respect to the total number of proteins (n= 573) in different intervals of log_2(fold change).

References

1. De Schryver, E. L. L. M., Design of ESPRIT: An international randomized trial for secondary prevention after non-disabling cerebral ischaemia of arterial origin. *Cerebrovasc. Dis.* **2000,** 10, (2), 147-150.
2. Halkes, P. H.; van Gijn, J.; Kappelle, L. J.; Koudstaal, P. J.; Algra, A., Aspirin plus dipyridamole versus aspirin alone after cerebral ischaemia of arterial origin (ESPRIT): randomised controlled trial. *Lancet* **2006,** 367, (9523), 1665-73.
3. Narasimhalu, K.; Ang, S.; De Silva, D. A.; Wong, M. C.; Chang, H. M.; Chia, K. S.; Auchus, A. P.; Chen, C. P., The prognostic effects of poststroke cognitive impairment no dementia and domain-specific cognitive impairments in nondisabled ischemic stroke patients. *Stroke* **2011,** 42, (4), 883-8.
4. Narasimhalu, K.; Ang, S.; De Silva, D. A.; Wong, M. C.; Chang, H. M.; Chia, K. S.; Auchus, A. P.; Chen, C., Severity of CIND and MCI predict incidence of dementia in an ischemic stroke cohort. *Neurology* **2009,** 73, (22), 1866-1872.
5. Bonita, R.; Beaglehole, R., Modification of rankin scale: Recovery of motor function after stroke. *Stroke* **1988,** 19, 1497–1500.
6. *Diagnostic and statistical manual of mental disorders-iv.* American Psychiatric Association, Washington, D.C., USA: 1994.
7. Mead, G. E.; Lewis, S. C.; Wardlaw, J. M.; Dennis, M. S.; Warlow, C. P., How well does the Oxfordshire community stroke project classification predict the site and size of the infarct on brain imaging? *J Neurol Neurosurg Psychiatry* **2000,** 68, (5), 558-62.
8. Datta, A.; Chen, C. P.; Sze, S. K., Discovery of Prognostic Biomarker Candidates of Lacunar Infarction by Quantitative Proteomics of Microvesicles Enriched Plasma *PLoS ONE* **2014,** 9, (4), e94663.
